# Supplementary material for: Generation of multitissue cell-cultivated meat via multidirectional differentiation of stable porcine epiblast stem cells
Source: Nat Commun. 2026 Mar 2;17:3347. doi: 10.1038/s41467-026-70177-w (PMC13066626; doi:10.1038/s41467-026-70177-w)
Supplement: Supplementary file 1 — Supplementary Information [file 41467_2026_70177_MOESM1_ESM.pdf]

Supplementary Information for

Generation of multitissue cell-cultivated meat via multidirectional differentiation of stable porcine epiblast stem cells

Yixuan Yao[1,*], Gaoxiang Zhu[1,*], Minglei Zhi[1,*], Runbo Li[1,*], Lun Qin[2], Yu Zhang[3], Yachun Chen[3], Xinze Chen[1], Tong Wang[1], Linzi Li[4], Yingjie Wang[1], Shunxin Wang[1], He Zhang[1], Xianchao Feng[4], Aijin Ma[3,**], Suying Cao[2,**], and Jianyong Han[1,**]

[1]State Key Laboratory of Animal Biotech Breeding, Frontiers Science Center for Molecular Design Breeding (MOE), China Agricultural University, Beijing 100193, People's Republic of China.

[2]Animal Science and Technology College, Beijing University of Agriculture, Beijing 102206, People's Republic of China.

[3]School of Food and Health, Beijing Technology and Business University, Beijing, China.

[4]College of Food Science and Engineering, Northwest A&F University, Yangling, Shanxi, China.

[*]These authors contributed equally: Yixuan Yao, Gaoxiang Zhu, Minglei Zhi, Runbo Li.

[**]e-mail: maaj@btbu.edu.cn; 20137602@bua.edu.cn; hanjy@cau.edu.cn

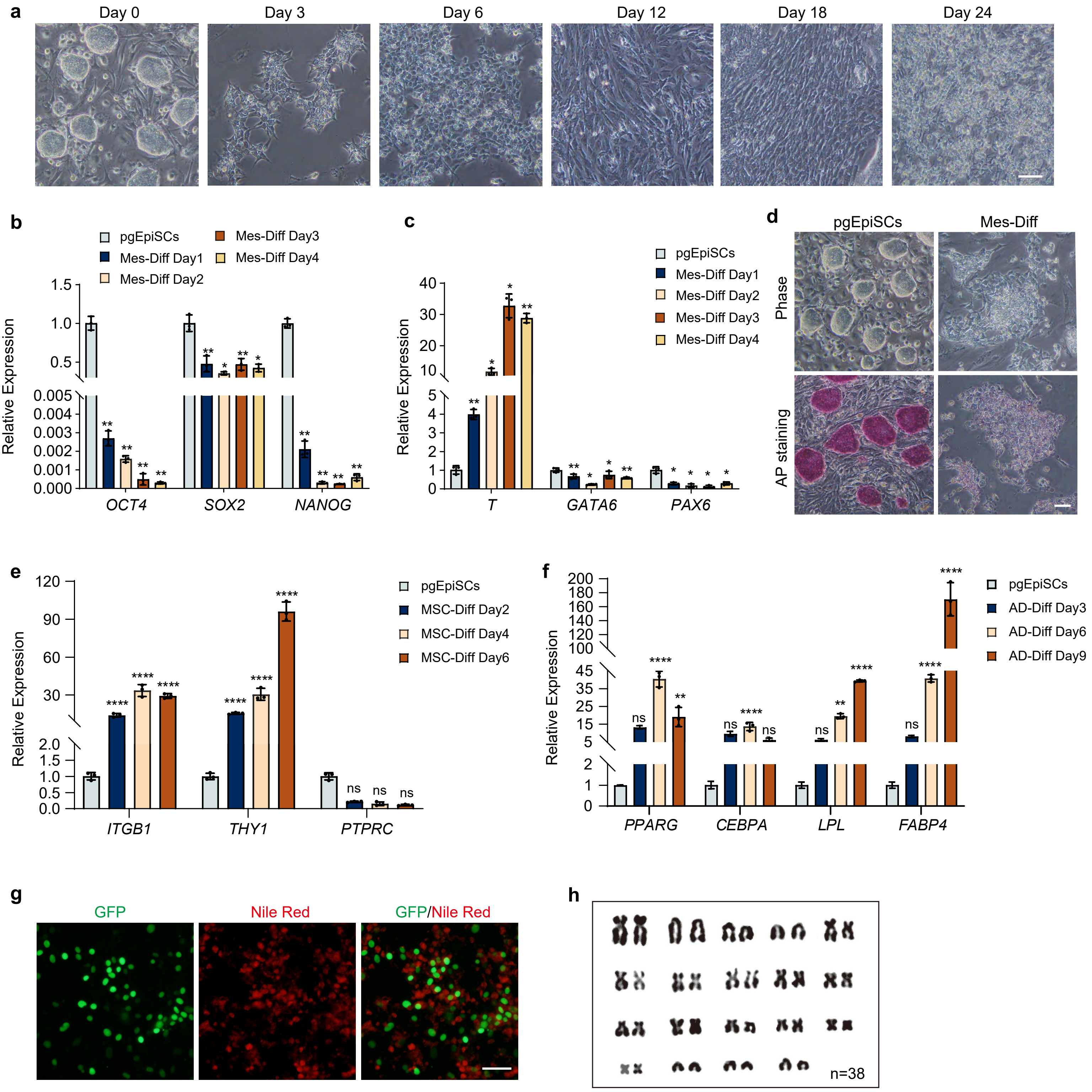
a
Day 0
Day 3
Day 6
Day 12
Day 18
Day 24
b
pgEpiSCs
Mes-Diff Day1
Mes-Diff Day2
Mes-Diff Day3
Mes-Diff Day4
Relative Expression
OCT4
SOX2
NANOG
c
pgEpiSCs
Mes-Diff Day1
Mes-Diff Day2
Mes-Diff Day3
Mes-Diff Day4
Relative Expression
T
GATA6
PAX6
d
pgEpiSCs
Mes-Diff
Phase
AP staining
e
pgEpiSCs
MSC-Diff Day2
MSC-Diff Day4
MSC-Diff Day6
Relative Expression
ITGB1
THY1
PTPRC
f
pgEpiSCs
AD-Diff Day3
AD-Diff Day6
AD-Diff Day9
Relative Expression
PPARG
CEBPA
LPL
FABP4
g
GFP
Nile Red
GFP/Nile Red
h
n=38

Supplementary Fig. 1 | Optimization of the culture system and identification of cell characteristics of pgEpiSCs during adipogenesis.

a, Cell morphology images at different stages of adipocyte differentiation. **b**, **c**, Expression of genes related to pluripotency (*OCT4, SOX2, NANOG*) and ectoderm (*PAX6*), mesoderm (*T* (*Brachyury*)), and endoderm (*GATA6*) in the process of differentiating into mesoderm from Day1 to Day4. Mes-Diff, pgEpiSCs differentiated into mesoderm. **d**, AP staining under the activation of WNT and the inhibition of TGF-β conditions. **e**, The expression of MSC-related genes *ITGB1, THY1* and *PTPRC* was tracked over time by qRT-PCR. MSCs-Diff, pgEpiSCs differentiated into MSCs. **f**, The expression of adipogenesis-related genes during the process of early (*PPARG*, *CEBPA*) or late (*LPL*, *FABP4*) adipogenic induction was tracked over time by qRT-PCR. ADs-Diff, pgEpiSCs differentiated into adipocytes. **g**, Adipogenic differentiation was observed by using the pgEpiSCs-NLS-GFP cell line. Nile red staining of GFP-cells, the GFP represents the nucleus and can be observed in single cell. **h**, Karyotype analysis of pgEpiSCs after adipogenic differentiation, which maintains normal chromosome numbers (n=38). For (**a**, **d**, **g**), Scale bar, 50 μm. For (**b**, **c**, **e**, **f**), error bars indicate means ± SD, n = 3. * $p < 0.05$, ** $p < 0.01$, *** $p < 0.001$, **** $p < 0.0001$, and ns indicates $p \geq 0.05$. Similar results were obtained in three independent experiments and represent significant using Two-way ANOVA, followed by Dunnett's multiple comparisons test. Exact *P* values are listed in Source Data Supplementary Fig. 1. Source data are provided as a Source Data file.

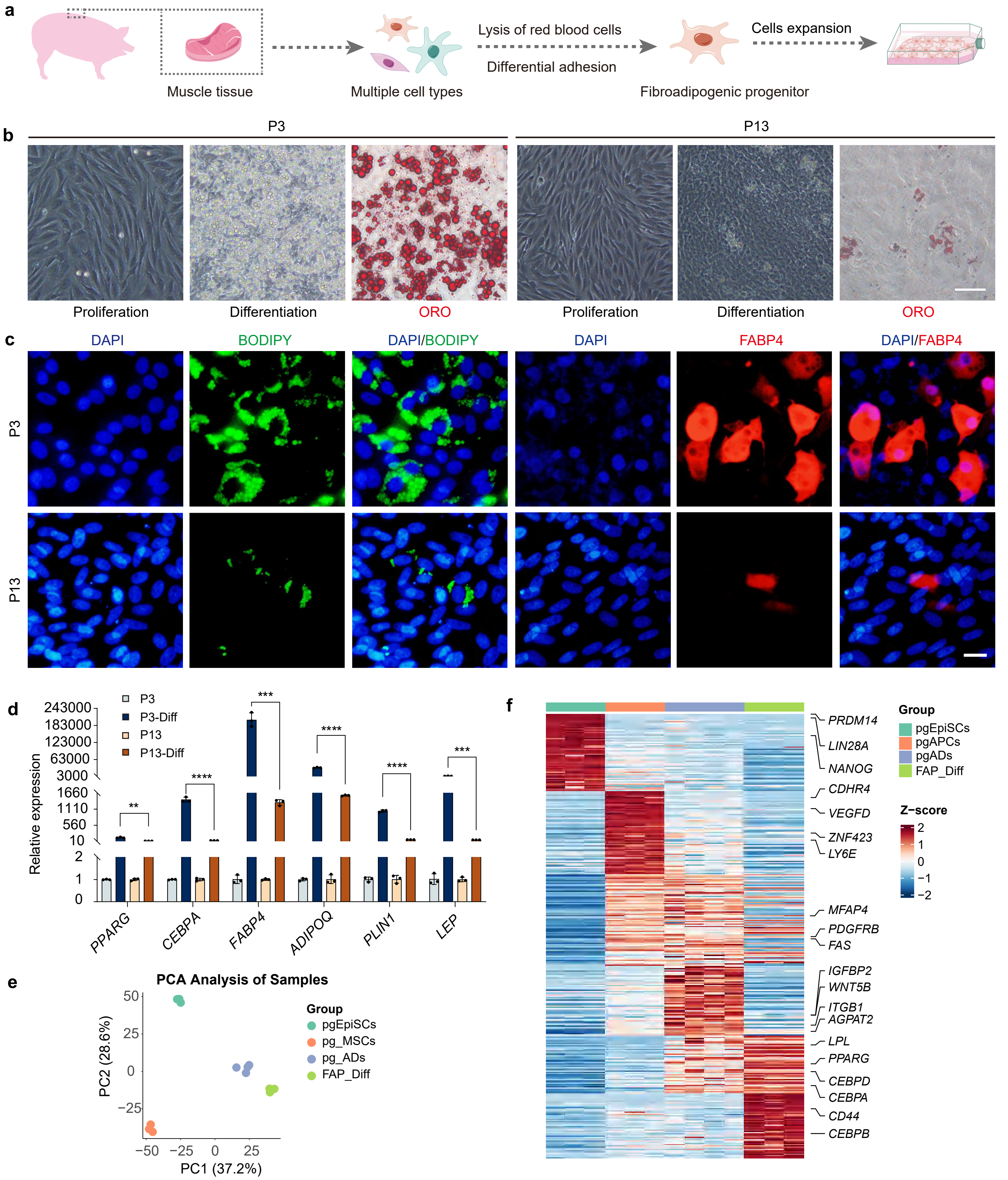

a
Muscle tissue
Multiple cell types
Lysis of red blood cells
Differential adhesion
Fibroadipogenic progenitor
Cells expansion
b
P3
P13
Proliferation
Differentiation
ORO
Proliferation
Differentiation
ORO
c
DAPI
BODIPY
DAPI/BODIPY
DAPI
FABP4
DAPI/FABP4
P3
P13
d
Relative expression
P3
P3-Diff
P13
P13-Diff
243000
183000
123000
63000
3000
1660
1110
560
10
2
1
0
PPARG
CEBPA
FABP4
ADIPOQ
PLIN1
LEP
**
****
***
****
****
***
e
PCA Analysis of Samples
PC2 (28.6%)
PC1 (37.2%)
Group
pgEpiSCs
pg_MSCs
pg_ADs
FAP_Diff
f
PRDM14
LIN28A
NANOG
CDHR4
VEGFD
ZNF423
LY6E
MFAP4
PDGFRB
FAS
IGFBP2
WNT5B
ITGB1
AGPAT2
LPL
PPARG
CEBPD
CEBPA
CD44
CEBPB
Group
pgEpiSCs
pgAPCs
pgADs
FAP_Diff
Z-score

Supplementary Fig. 2 | Identification of differentiation potential of Porcine fibro/adipogenic progenitors (pFAPs)

a, Schematic diagram of pFAPs isolation. **b,** Morphology and Oil Red O Staining of proliferation and differentiation of pFAPs and for different passages. Scale bar, 50 μm. **c**, BODIPY staining of LDs and immunostaining of FABP4 of adipocyte differentiated from P3 and P13. Green, BODIPY; red, FABP4; blue, DAPI. Scale bar, 20 μm. **d,** Expression of genes related to adipocyte differentiation of *PPARG, CEBPA, FABP4, ADIPOQ, PLIN1* and *LEP* by qRT-PCR from P3 and P13. Error bars indicate means ± SD, n = 3. ** $p < 0.01$, *** $p < 0.001$, **** $p < 0.0001$, similar results were obtained in three independent experiments and represent significant using two-tailed student's *t* test. Exact *P* values are listed in Source Data Supplementary Fig. 2. P3-Diff, P3-pFAPs differentiated into adipocytes. P13-Diff, P13-pFAPs differentiated into adipocytes. **e**, PCA plot of four populations from the adipogenesis of pgEpiSCs *in vitro* and ADs derived from P3-pFAPs. The different colors indicate different cell populations. **f**, Heatmap of DEGs between differentiation of pFAPs isolated *in vivo* and pgAPCs and Ads. For (**b**-**d**), similar results were obtained in three independent experiments. Source data are provided as a Source Data file.

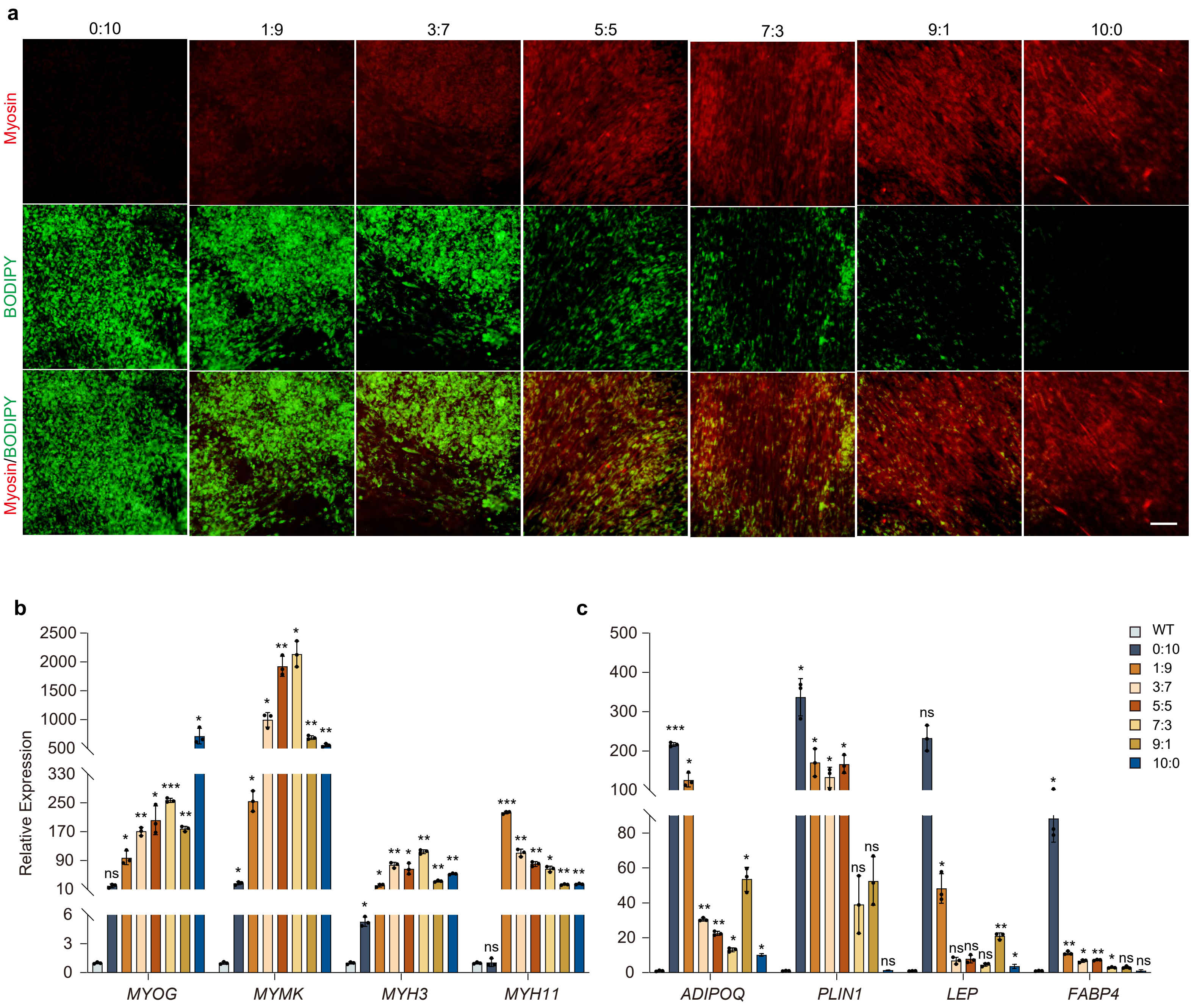

a
0:10
1:9
3:7
5:5
7:3
9:1
10:0
Myosin
BODIPY
Myosin/BODIPY
b
Relative Expression
2500
2000
1500
1000
500
330
250
170
90
10
6
3
0
MYOG
MYMK
MYH3
MYH11
c
500
400
300
200
100
80
60
40
20
0
ADIPOQ
PLIN1
LEP
FABP4
WT
0:10
1:9
3:7
5:5
7:3
9:1
10:0

Supplementary Fig. 3 | Myoblasts and preadipocytes derived from pgEpiSCs could be co-cultured in different proportions.

a, Fluorescence staining with BODIPY and Myosin was used to assess the lipid droplets and muscle fiber content in co-cultured cells with varying myoblast-to-preadipocyte ratios. Scale bar, 50 μm. **b**, **c**, Relative mRNA expression levels of muscle-related genes (*MYOG, MYMK, MYH3, MYH11*) and adipocyte-related genes (*ADIPOQ, PLIN1, LEP, FABP4*) were analyzed via qRT-PCR. Error bars indicate the means ± SDs, (n = 3). *$p < 0.05$, **$p < 0.01$, ***$p < 0.001$, and ns ($p > 0.05$), similar results were obtained in three independent experiments and represent significant using Two-way ANOVA, followed by Dunnett's multiple comparisons test. Exact *P* values are listed in Source Data Supplementary Fig. 3. WT, undifferentiated pgEpiSCs. (0:10, 1:9, 3:7, 5:5, 7:3, 9:1, 10:0) pgEpiSCs-myoblast and preadipocyte were cocultured at a ratio of 0:10, 1:9, 3:7, 5:5, 7:3, 9:1, 10:0. Source data are provided as a Source Data file.

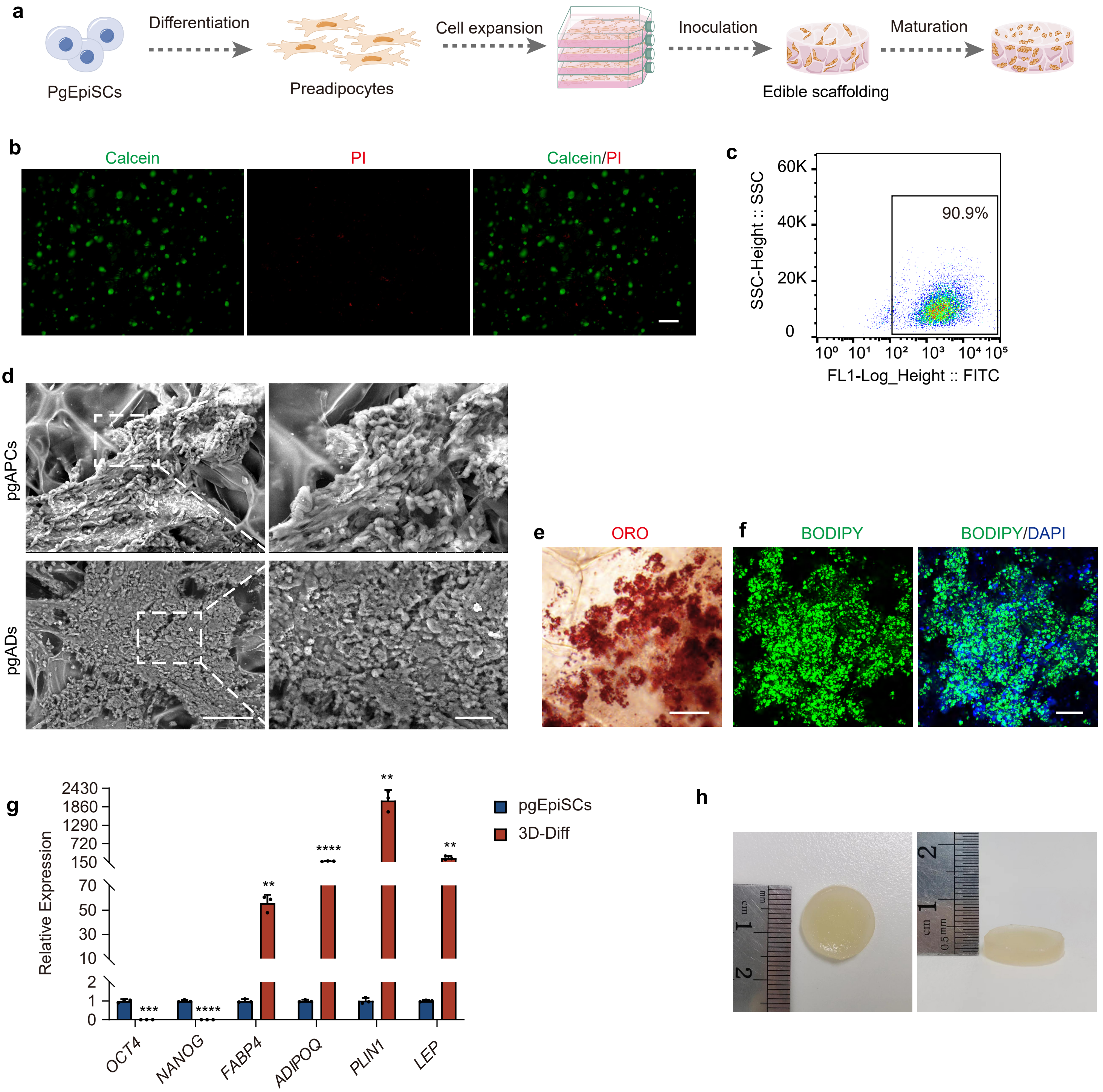
a
Differentiation
Cell expansion
Inoculation
Maturation
PgEpiSCs
Preadipocytes
Edible scaffolding
b
Calcein
PI
Calcein/PI
c
SSC-Height :: SSC
FL1-Log_Height :: FITC
90.9%
d
pgAPCs
pgADs
e
ORO
f
BODIPY
BODIPY/DAPI
g
Relative Expression
pgEpiSCs
3D-Diff
OCT4
NANOG
FABP4
ADIPOQ
PLIN1
LEP
h

Supplementary Fig. 4 | pgEpiSCs-derived preadipocytes attachment and maturation on plant-based edible 3D scaffolds.

a, Schematic illustration of pgEpiSCs-preadipocyte seeding onto a 3D scaffold. **b**, Live/dead cell staining using Calcein-AM (green) and PI (red) 24 h after seeding revealed high cell viability and negligible cell death. Scale bar, 100 μm. **c**, Flow cytometry analysis demonstrated high cell viability on 3D scaffolds at 24 h post-seeding. **d**, Scanning electron microscopy (SEM) images showing the cellular architecture of pgEpiSCs-preadipocytes on scaffolds before and after 10 days of adipogenic induction. Scale bars, 50 μm (left) and 20 μm (right). **e**, **f**, Oil Red O and BODIPY staining demonstrated the accumulation and maturation of LDs in adipogenically differentiated pgEpiSCs-preadipocytes on scaffolds. Scale bar, 50 μm. **g**, Quantification of mRNA expression of pluripotency (*OCT4*, *NANOG*) and maturation of adipogenic differentiation (*FABP4*, *ADIPOQ*, *LEP*, *PLIN1*) related genes by qRT-PCR. Error bars indicate means ± SD, n = 3. ** $p < 0.01$, **** $p < 0.0001$, similar results were obtained in three independent experiments and represent significant using two-tailed student's *t* test. Exact *P* values are listed in Source Data Supplementary Fig. 4. 3D-Diff, pgEpiSCs differentiated into adipose on 3D scaffolds. **h**, Appearance of pgEpiSCs-derived CM with scaffold in culture for 10d. Source data are provided as a Source Data file.

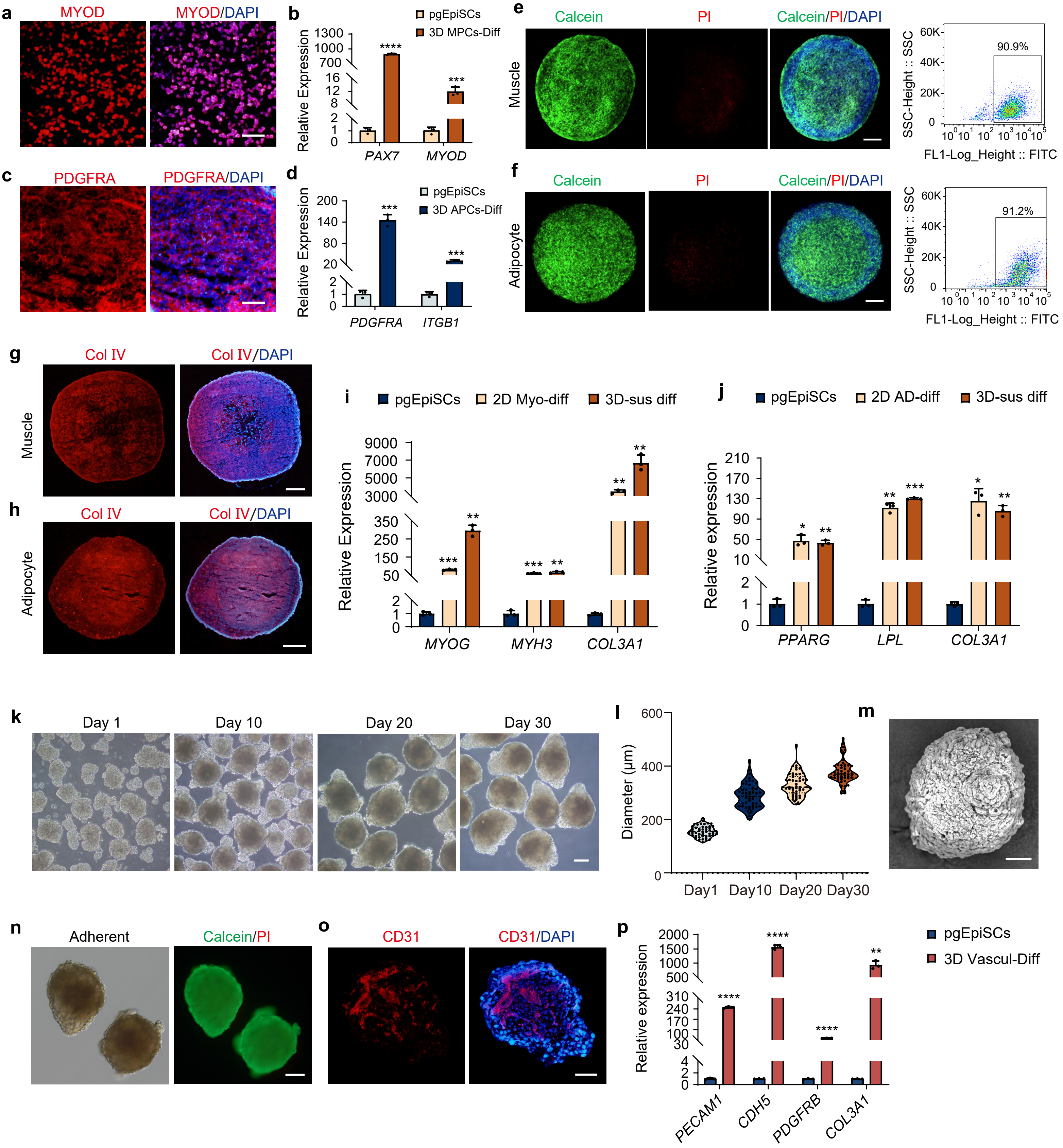

a
MYOD
MYOD/DAPI
b
pgEpiSCs
3D MPCs-Diff
Relative Expression
PAX7
MYOD
c
PDGFRA
PDGFRA/DAPI
d
pgEpiSCs
3D APCs-Diff
Relative Expression
PDGFRA
ITGB1
e
Calcein
PI
Calcein/PI/DAPI
Muscle
90.9%
SSC-Height :: SSC
FL1-Log_Height :: FITC
f
Adipocyte
91.2%
g
Col IV
Col IV/DAPI
Muscle
h
Adipocyte
i
pgEpiSCs
2D Myo-diff
3D-sus diff
Relative Expression
MYOG
MYH3
COL3A1
j
pgEpiSCs
2D AD-diff
3D-sus diff
Relative expression
PPARG
LPL
COL3A1
k
Day 1
Day 10
Day 20
Day 30
l
Diameter (μm)
Day1
Day10
Day20
Day30
m
n
Adherent
Calcein/PI
o
CD31
CD31/DAPI
p
pgEpiSCs
3D Vascul-Diff
Relative expression
PECAM1
CDH5
PDGFRB
COL3A1

Supplementary Fig. 5 | Characterization of spheroids derived from progenitor cells.

a, Imaging of MYOD in cross sections of pgMPCs-derived spheroids at day 3. **b**, Analysis of *PAX7* and *MYOD* expression in pgMPCs spheroids. MPCs-Diff, pgEpiSCs differentiated into MPCs. **c**, Imaging of PDGFRA of pgAPCs-derived spheroids at day 3. **d,** Analysis of *ITGB1* and *PDGFRA* expression in pgAPCs spheroids. APCs-Diff pgEpiSCs differentiated into APCs. **e, f,** Fluorescence image and flow cytometry analysis of **e,** muscle-spheroid or **f,** adipose-spheroid stained with Calcein-AM and PI. **g, h,** Representative fluorescence images of collagen formation (Col IV) in muscle or adipose spheroids. **i,** Quantification of mRNA expression of myogenesis (*MYOG*, *MYH3*) and ECM formation (*COL3A1*) related genes between 2D and 3D suspension myogenic differentiation. 2D Myo-Diff, muscle cells derived from pgEpiSCs in 2D system. 3D-sus diff, muscle-spheroids derived from pgEpiSCs in 3D suspension system. **j,** Gene expression of adipogenesis (*PPARG, LPL*) and ECM formation (*COL3A1*) between 2D and 3D suspension adipogenic differentiation. 2D AD-Diff terminally differentiated adipose derived from pgEpiSCs in 2D system. 3D-sus diff, adipose-spheroids derived from pgEpiSCs in 3D suspension system. **k,** Bright field images of vascular spheroids from pgEpiSCs during the 30-day cultivation process. **l,** The diameter statistics of the vascular spheroids from pgEpiSCs within the 30-day culture. **m,** SEM images of vascular spheroids. **n,** Live/dead staining of pgEpiSCs-vascular spheroids. **o,** CD31 staining of pgEpiSCs-vascular spheroids. **p,** Gene expression of *PECAM1, CDH5, PDGFRB* and *COL3A1* by qPCR from pgEpiSCs-vascular spheroids. 3D Vascul-Diff, vascular-spheroids derived from pgEpiSCs in 3D suspension system. For (**c**, **d, o**), Scale bar, 50 μm. For (**e-h, k, m, n**), Scale bar, 100 μm. For (**b, d, l, j, n**) Error bars indicate means ± SD, n = 3. * $p < 0.1$, ** $p < 0.01$, *** $p < 0.001$, **** $p < 0.0001$. Two-tailed student's *t* test was used to analyze in **b**, **d**, **p**. Two-way ANOVA, followed by Dunnett's multiple comparisons test was used in **i**, **j**. Similar results were obtained in three independent experiments. Exact *P* values are listed in Source Data Supplementary Fig. 5. Source data are provided as a Source Data file.

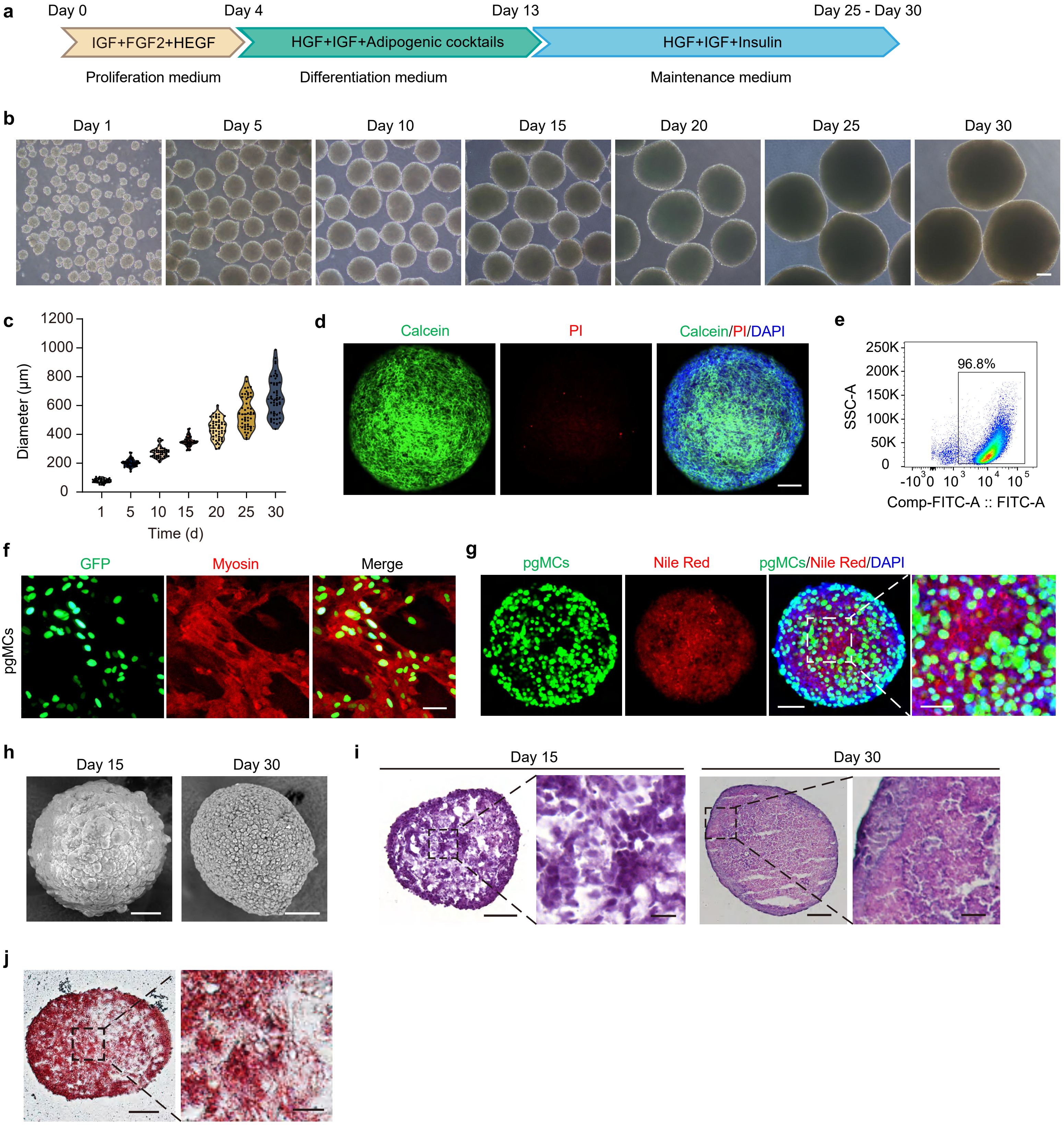

a
Day 0
Day 4
Day 13
Day 25 - Day 30
IGF+FGF2+HEGF
HGF+IGF+Adipogenic cocktails
HGF+IGF+Insulin
Proliferation medium
Differentiation medium
Maintenance medium
b
Day 1
Day 5
Day 10
Day 15
Day 20
Day 25
Day 30
c
Diameter (μm)
Time (d)
d
Calcein
PI
Calcein/PI/DAPI
e
96.8%
SSC-A
Comp-FITC-A :: FITC-A
f
GFP
Myosin
Merge
pgMCs
g
pgMCs
Nile Red
pgMCs/Nile Red/DAPI
h
Day 15
Day 30
i
Day 15
Day 30
j

Supplementary Fig. 6 | The formation process of muscle-adipose spheroids from pgMPCs and pgAPCs.

a, 3D aggregation scheme for pgMPCs and pgAPCs. The initial cell density was 5×10^5 cells / mL and cultured in a shaker for 30 days. **b**, Light microscopic images of muscle-adipose spheroids forming from day 1 to day 30. Scale bar, 100 μm. **c**, The diameter statistics of 3D suspension differentiation muscle-adipose spheroids, n =50, the violin plot is cut at the lowest and highest values, dotted line: quartile, dashed line: median, and dots represent single data points. **d, e,** Fluorescence image and flow cytometry analysis of muscle-adipose spheroids stained with Calcein-AM (green) and PI (red) and after 3D suspension culture on day 30. Scale bar, 100 μm. **f,** Establishment of pgEpiSCs-NLS-GFP fluorescent reporter cell lines and characterization of the myogenic differentiation process. Myosin is marker for muscle fibers. Scale bar, 50 μm. **g**, Characterization of muscle-adipose spheroids at day 30. Green represents pgMCs, Nile Red (red) represents pgADs. Scale bar, 100 μm (left) and 50 μm (right).**h**, SEM observation of spheroids forming from day 15 and day 30. Scale bar, 100 μm (left), and 200 μm (right). **i**, HE staining of muscle-adipose spheroid forming from day 15 and day 30. Scale bar, 100 μm (left), and 20 μm (right). **j**, Oil Red O staining of muscle-adipose spheroid forming from day 30. Scale bar, 100 μm (left), and 20 μm (right). For (**b-j)**, similar results were obtained in three independent experiments. Source data are provided as a Source Data file.

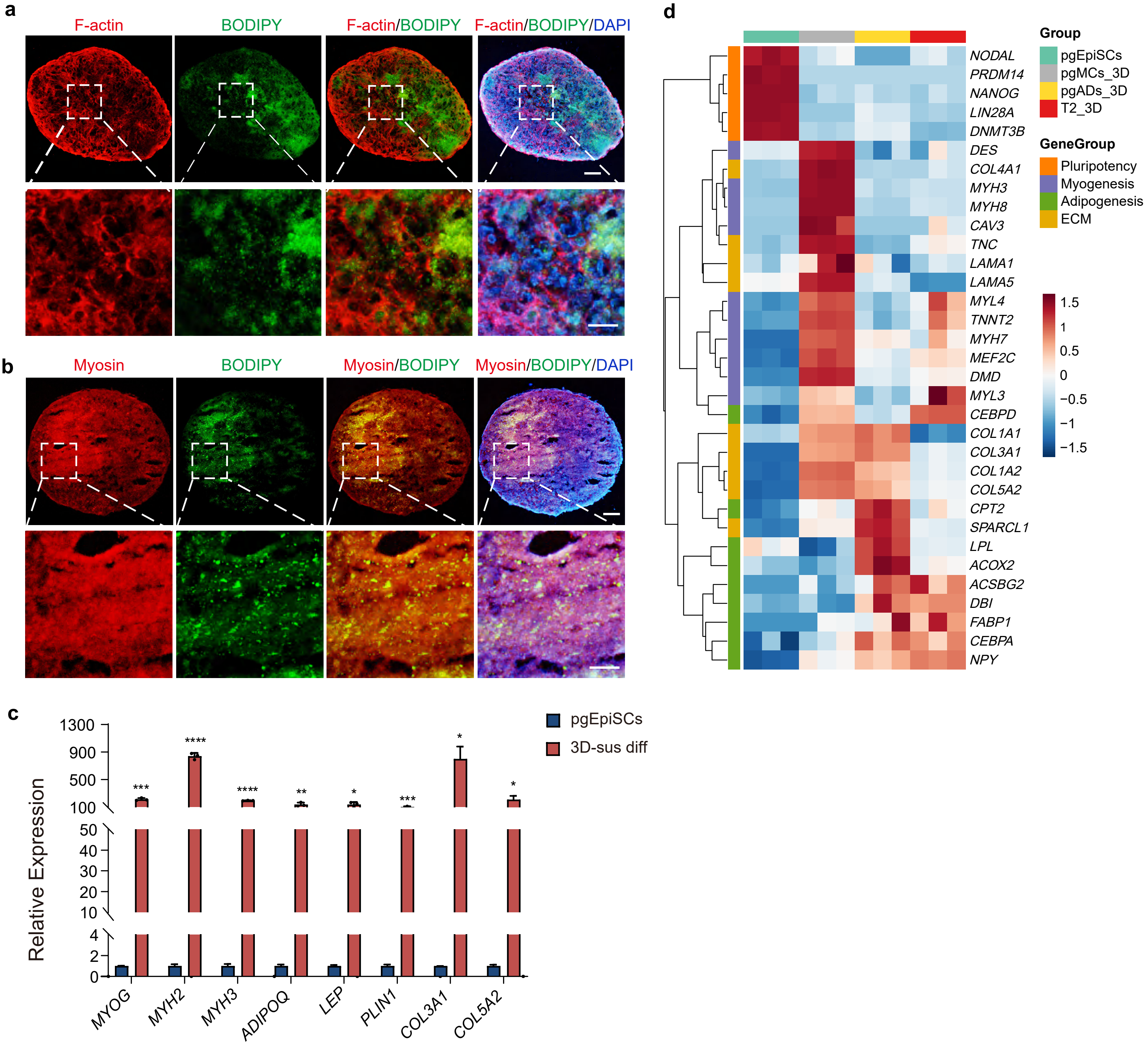

a
F-actin
BODIPY
F-actin/BODIPY
F-actin/BODIPY/DAPI
b
Myosin
BODIPY
Myosin/BODIPY
Myosin/BODIPY/DAPI
c
Relative Expression
1300
900
500
100
50
40
30
20
10
4
2
0
pgEpiSCs
3D-sus diff
***
****
****
**
*
***
*
*
MYOG
MYH2
MYH3
ADIPOQ
LEP
PLIN1
COL3A1
COL5A2
d
Group
pgEpiSCs
pgMCs_3D
pgADs_3D
T2_3D
GeneGroup
Pluripotency
Myogenesis
Adipogenesis
ECM
1.5
1
0.5
0
−0.5
−1
−1.5
NODAL
PRDM14
NANOG
LIN28A
DNMT3B
DES
COL4A1
MYH3
MYH8
CAV3
TNC
LAMA1
LAMA5
MYL4
TNNT2
MYH7
MEF2C
DMD
MYL3
CEBPD
COL1A1
COL3A1
COL1A2
COL5A2
CPT2
SPARCL1
LPL
ACOX2
ACSBG2
DBI
FABP1
CEBPA
NPY

Supplementary Fig. 7 | Characteristics of self-assembling muscle-adipose spheroids.

a, **b**, Representative fluorescence images of muscle fibers (Myosin), LDs (BODIPY) and cytoskeleton (F-actin) of the cross-section of the spheroids in day30. Scale bars, 50 μm (above) and 20 μm (below). **c**, Quantitative RT-PCR analysis of mRNA expression levels of myogenic differentiation (*MYOG*, *MYH2*, *MYH3*), adipogenic differentiation (*ADIPOQ*, *PLIN1*, *LEP*), and collagen formation (*COL3A1*, *COL5A2*) related genes. Error bars represent mean ± SD (n = 3). *$p < 0.1$, **$p < 0.01$, ***$p < 0.001$, ****$p < 0.0001$, similar results were obtained in three independent experiments and represent significant using two-tailed student's *t* test. Exact *P* values are listed in Source Data Supplementary Fig. 7. 3D-sus diff, muscle-adipose spheroids derived from pgEpiSCs in 3D suspension system. **d**, Heatmap showing the difference in genes expression associated with pluripotency, myogenesis, adipogenesis and ECM formation in muscle, adipose and muscle-adipose spheroids. pgMCs_3D, muscle-spheroids formed within 3D suspension culture system. pgADs_3D, adipose-spheroids formed within 3D suspension culture system. T2_3D, spheroids derived from two different cell type sources. Source data are provided as a Source Data file.

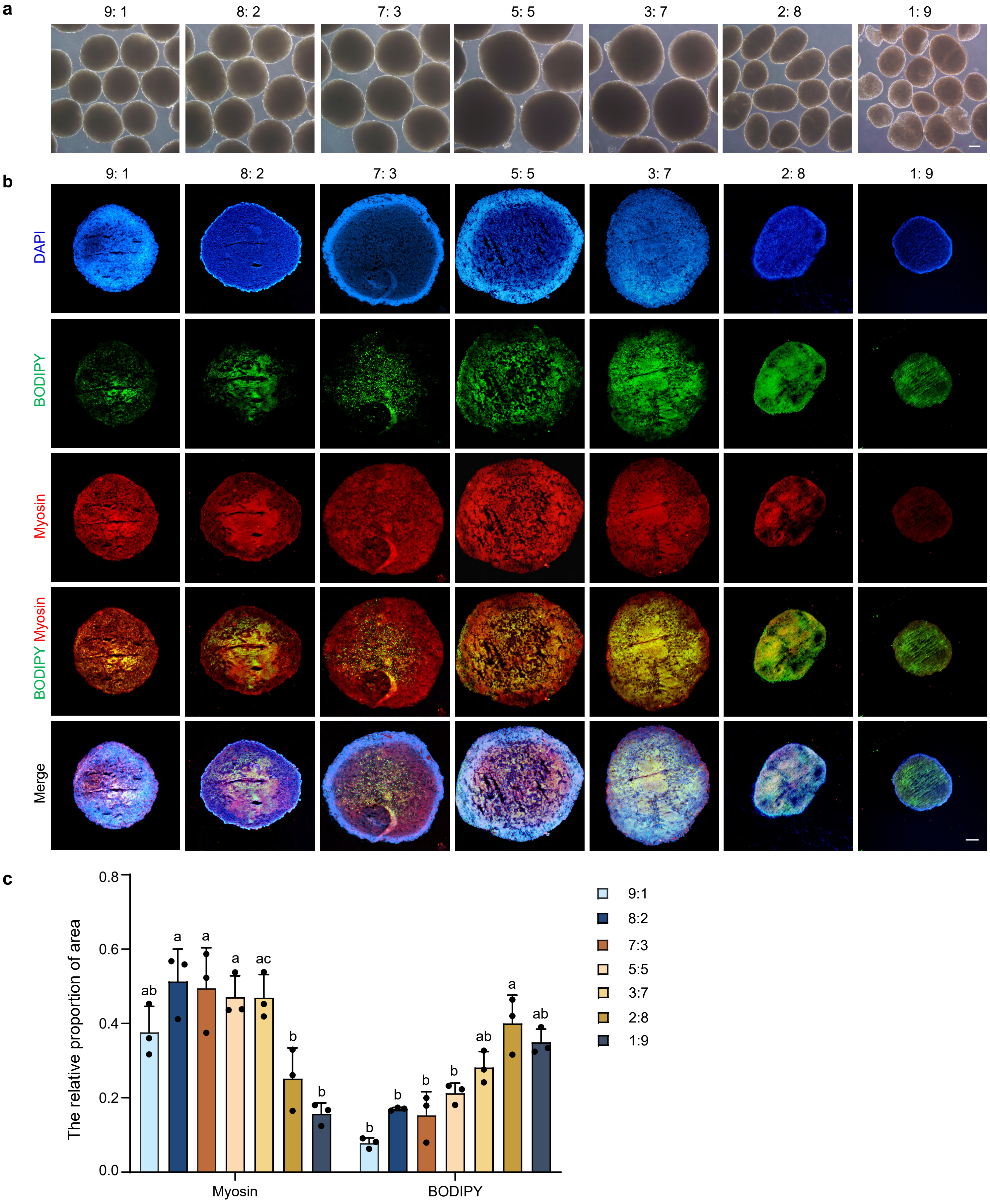
a
9: 1
8: 2
7: 3
5: 5
3: 7
2: 8
1: 9
b
9: 1
8: 2
7: 3
5: 5
3: 7
2: 8
1: 9
DAPI
BODIPY
Myosin
BODIPY Myosin
Merge
c
The relative proportion of area
0.0
0.2
0.4
0.6
0.8
ab
a
a
a
ac
b
b
b
b
b
b
ab
a
ab
Myosin
BODIPY
9:1
8:2
7:3
5:5
3:7
2:8
1:9

Supplementary Fig. 8 | Characteristics of muscle- adipose spheroids composed from different seeding cell ratios of pgMPCs and pgAPCs.

a, Brightfield of muscle-adipose spheroids composed with different ratios of pgMPCs and pgAPCs. Scale bar, 200 μm. **b**, Fluorescence staining with BODIPY and Myosin was used to assess the lipid droplets and muscle fiber content in muscle-adipose spheroids with varying pgMPCs- to -pgAPCs ratios. Scale bar, 200 μm. **c**, Relative proportion of area of BODIPY and Myosin from muscle-adipose spheroids with varying pgMPCs- to -pgAPCs ratios. Error bars indicate means ± SD, n = 3. Data was analyzed using a Two-way ANOVA, followed by Bonferroni's multiple comparisons test and different letters represent significant differences at $p < 0.05$, similar results were obtained in three independent experiments. (9:1, 8:2, 7:3, 5:5, 3:7, 2:8, 1:9) muscle- adipose spheroids composed from pgMPCs and pgAPCs at a ratio of 9:1, 8:2, 7:3, 5:5, 3:7, 2:8, 1:9. Exact *P* values are listed in Source Data Supplementary Fig. 8. Source data are provided as a Source Data file.

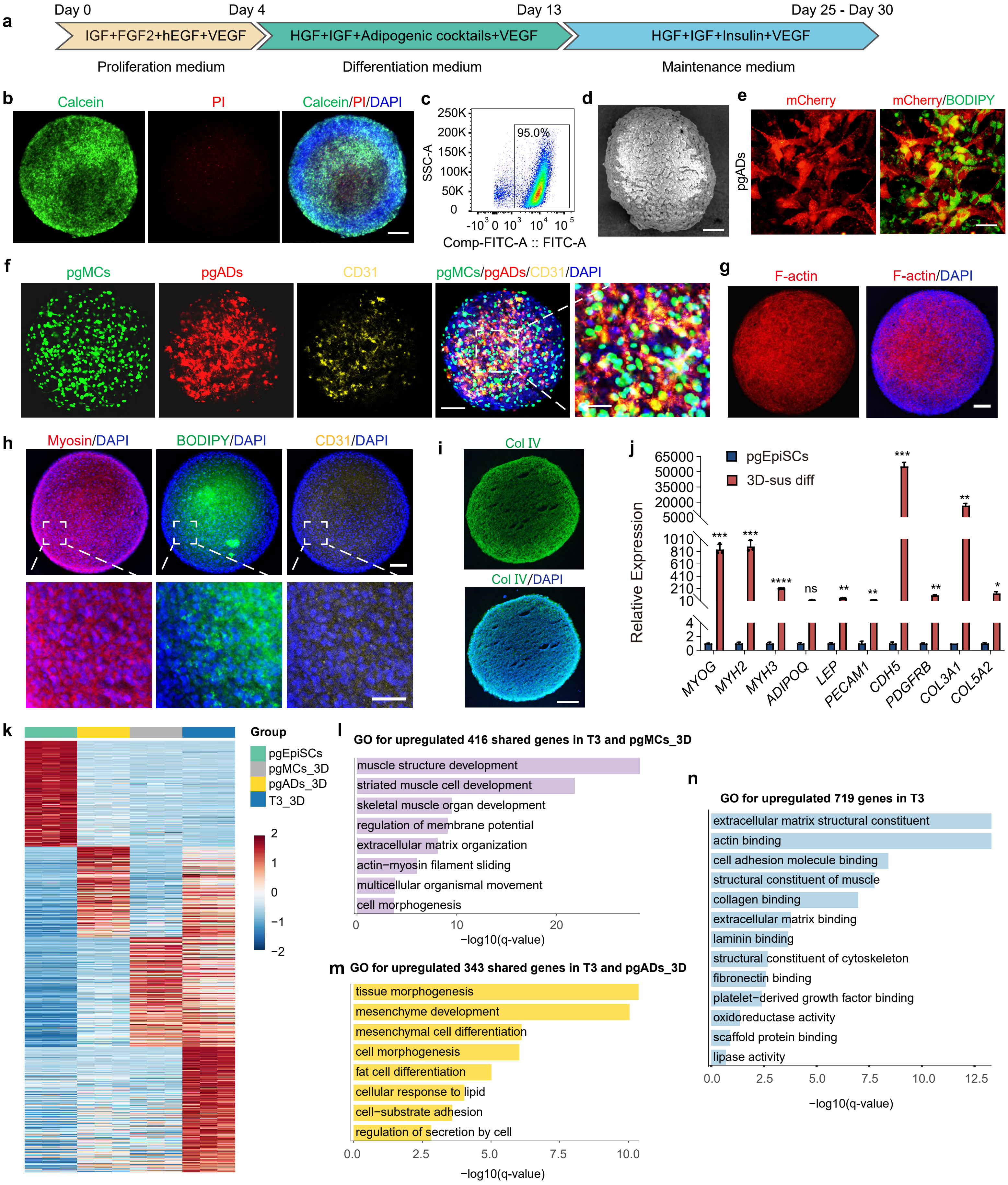
a
Day 0
Day 4
Day 13
Day 25 - Day 30
IGF+FGF2+hEGF+VEGF
HGF+IGF+Adipogenic cocktails+VEGF
HGF+IGF+Insulin+VEGF
Proliferation medium
Differentiation medium
Maintenance medium
b
Calcein
PI
Calcein/PI/DAPI
c
SSC-A
250K
200K
150K
100K
50K
0
95.0%
Comp-FITC-A :: FITC-A
d
e
pgADs
mCherry
mCherry/BODIPY
f
pgMCs
pgADs
CD31
pgMCs/pgADs/CD31/DAPI
g
F-actin
F-actin/DAPI
h
Myosin/DAPI
BODIPY/DAPI
CD31/DAPI
i
Col IV
Col IV/DAPI
j
pgEpiSCs
3D-sus diff
Relative Expression
MYOG
MYH2
MYH3
ADIPOQ
LEP
PECAM1
CDH5
PDGFRB
COL3A1
COL5A2
***
****
ns
**
*
k
Group
pgEpiSCs
pgMCs_3D
pgADs_3D
T3_3D
l
GO for upregulated 416 shared genes in T3 and pgMCs_3D
muscle structure development
striated muscle cell development
skeletal muscle organ development
regulation of membrane potential
extracellular matrix organization
actin−myosin filament sliding
multicellular organismal movement
cell morphogenesis
−log10(q-value)
m
GO for upregulated 343 shared genes in T3 and pgADs_3D
tissue morphogenesis
mesenchyme development
mesenchymal cell differentiation
cell morphogenesis
fat cell differentiation
cellular response to lipid
cell−substrate adhesion
regulation of secretion by cell
−log10(q-value)
n
GO for upregulated 719 genes in T3
extracellular matrix structural constituent
actin binding
cell adhesion molecule binding
structural constituent of muscle
collagen binding
extracellular matrix binding
laminin binding
structural constituent of cytoskeleton
fibronectin binding
platelet−derived growth factor binding
oxidoreductase activity
scaffold protein binding
lipase activity
−log10(q-value)

Supplementary Fig. 9 | Characteristic identification of multi-organizational 3D self-assembling spheroids.

a, 3D aggregation scheme for pgMPCs, pgAPCs, and pgVPCs. **b**, Live/dead cell staining with Calcein-AM and PI of muscle-adipose-vascular spheroids during 3D suspension culture from day 30. Scale bar, 100 μm. **c**, Flow cytometry analysis demonstrated high cell viability on muscle-adipose-vascular spheroids after Live/dead cell staining. **d**, SEM images of spheroids formed at day 30. Scale bar, 100 μm. **e,** Adipogenic differentiation of pgEpiSCs-mCherry fluorescent reporter cell lines and characterization of pgADs. Scale bar, 50 μm. **f,** Characterization of muscle-adipose-vascular spheroids at day 30. Green represents pgMCs, red represents pgADs, yellow (CD31) represents pgECs. Scale bar, 100 μm (left) and 50 μm (right). **g,** F-actin staining of the 1: 1: 1 spheroid, Scale bar, 100 μm; **h**, Confocal imaging of the staining of the 1:1:1 spheroid. Scale bar, 100 μm (above) and 50 μm (blow). **i**, Fluorescence images showed collagen formation (Col IV) in muscle-adipose-vascular spheroids. Scale bar, 100 μm. **j**, qRT-PCR analysis of mRNA expression levels of myogenesis (*MYOG*, *MYH2*, *MYH3*), adipogenesis (*ADIPOQ*, *PLIN1*, *LEP*), vasculogenesis (*PECAM1*, *CDH5*, *PDGFRB*) and collagen formation (*COL3A1*, *COL5A2*) related genes. 3D-sus diff, muscle-adipose-vascular spheroids derived from pgEpiSCs in 3D suspension system. **k**, Heatmap illustrating the expression of T3_3D-specific genes and genes shared with pgMCs_3D or pgADs_3D groups, highlighting the multi-lineage characteristics of T3_3D cells. **l**, **m**, GO enrichment analysis of genes upregulated in both T3_3D and pgMCs_3D, emphasizing shared (**l**), myogenic and (**m**), adipogenic differentiation-related pathways. **n**, GO enrichment analysis of genes upregulated in T3_3D compared to other groups, highlighting T3-specific biological pathways. pgMCs_3D, muscle-spheroids formed within 3D suspension culture system. pgADs_3D, adipose-spheroids formed within 3D suspension culture system. T3_3D, spheroids derived from three different cell type sources. For **j**, error bars represent mean ± SD (n = 3). *$p < 0.1$, **$p < 0.01$, ***$p < 0.001$, ****$p < 0.0001$, ns indicates $p \geq 0.05$. similar results were obtained in three independent experiments and represent significant using two-tailed student's *t* test. Exact *P* values are listed in Source Data Supplementary Fig. 9. Source data are provided as a Source Data file.

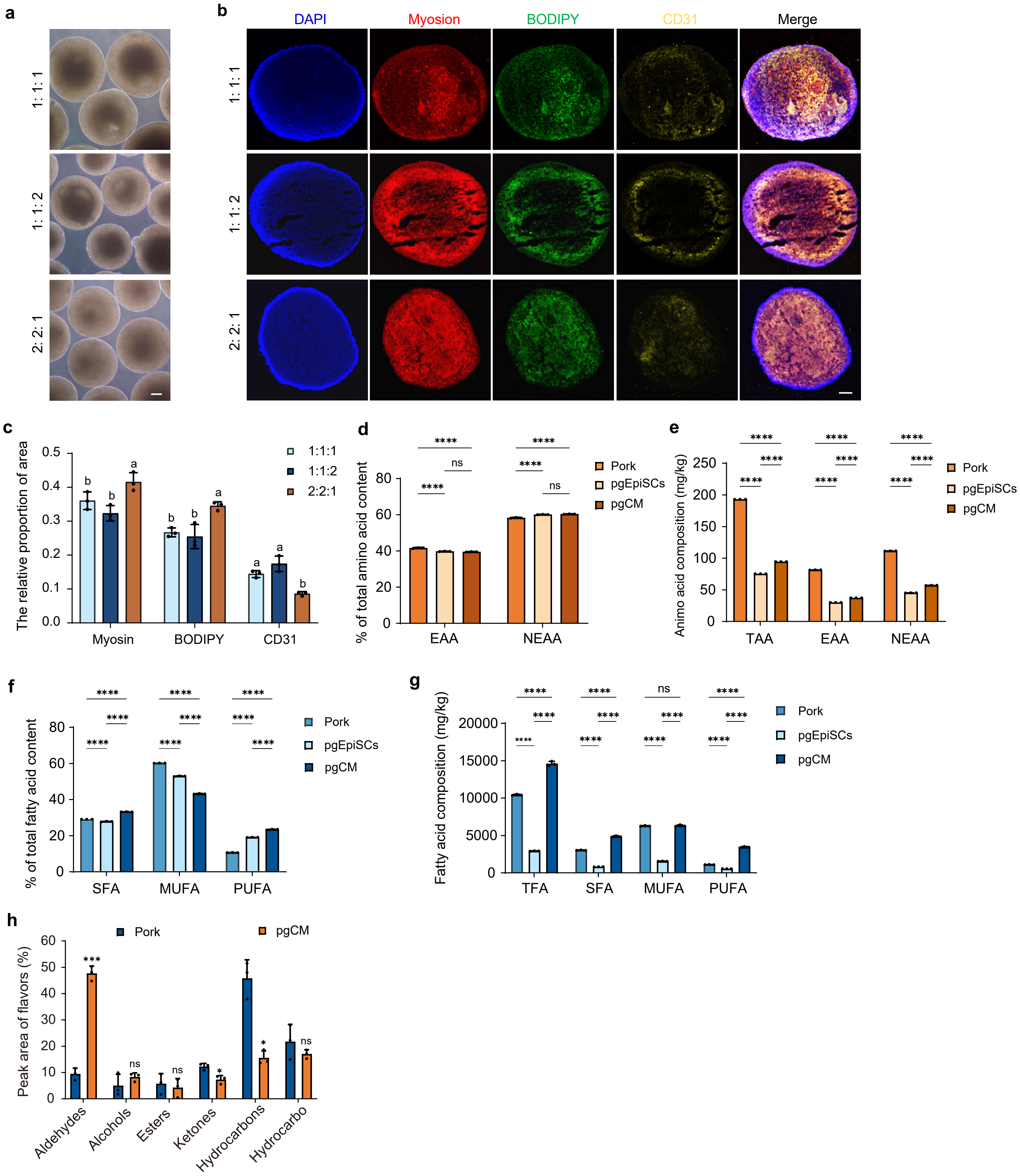

a
1: 1: 1
1: 1: 2
2: 2: 1
b
DAPI
Myosion
BODIPY
CD31
Merge
c
The relative proportion of area
1:1:1
1:1:2
2:2:1
Myosin
BODIPY
CD31
d
% of total amino acid content
Pork
pgEpiSCs
pgCM
EAA
NEAA
e
Animo acid composition (mg/kg)
TAA
EAA
NEAA
f
% of total fatty acid content
SFA
MUFA
PUFA
g
Fatty acid composition (mg/kg)
TFA
SFA
MUFA
PUFA
h
Peak area of flavors (%)
Pork
pgCM
Aldehydes
Alcohols
Esters
Ketones
Hydrocarbons
Hydrocarbo

Supplementary Fig. 10 | Tissue and nutritional composition of multi-tissue spheroids.

a, Brightfield of muscle-adipose-vascular spheroids composed with different ratios of pgMPCs, pgAPCs and pgVPCs. Scale bar, 200 μm. **b**, Fluorescence staining with BODIPY, Myosin and CD31 was used to assess the lipid droplets, muscle fibers and ECs content in muscle-adipose-vescular spheroids with varying pgMPCs- to -pgAPCs to - pgVPCs ratios. Scale bar, 200 μm. **c**, Relative proportion of area of BODIPY, Myosin and CD31 from muscle-adipose-vascular spheroids with varying seeding cell ratios. Error bars indicate means ± SD, n = 3, different letters represent significant differences at $p < 0.05$. (2: 2: 1, 1: 1: 1, 1: 1: 2) muscle- adipose- vescular spheroids composed from pgMPCs, pgAPCs and pgVCs at a ratio of 2: 2: 1, 1: 1: 1, 1: 1: 2. **d**, Overview of essential (EAA) and nonessential (NEAA) amino acids in spheroids and conventional pork**.** **e**, Comparative analysis of amino acid composition about pgEpiSCs, multi-tissue spheroids and conventional pork. **f**, Overview of saturated (SFA), monounsaturated (MUFA) and polyunsaturated (PUFA) fatty acids in spheroids and conventional pork. **g**, Detailed comparison of fatty acid composition about pgEpiSCs, multi-tissue spheroids and conventional pork. **h**, The composition of flavor compounds of pgCM and pork cultured sausages. For **d**-**h**, Error bars represent mean ± SD (n = 3). *$p < 0.1$, ***$p < 0.001$, ****$p < 0.0001$, ns indicates $p \geq 0.05$. Two-way ANOVA, followed by Bonferroni's multiple comparisons test was used in **c**, **d**-**g**, and Two-tailed student's *t* test was used to analyze in **h**. Exact *P* values are listed in Source Supplementary Data Fig. 10. Similar results were obtained in three independent experiments. Source data are provided as a Source Data file.

Supplementary information, Key Resources Table.

Supplementary Table 1 Chemicals, Peptides and Recombinant Protein		
**REAGENT or RESOURCE**	**SOURCE**	**IDENTIFIER**
CHIR99021	Selleckchem	Cat# S1263
IWR-1-endo	Selleckchem	Cat# S7086
Y-27632	Selleckchem	Cat# S1049
WH-4-023	Selleckchem	Cat# S7565
Recombinant Human LIF	PeproTech	Cat# 300-05
Human/Murine/Rat Activin A	PeproTech	Cat# 120-14E
Recombinant Human FGF-2	PeproTech	Cat# 100-18B
Recombinant Human IGF-1	PeproTech	Cat# 100-11
Recombinant Human HGF	PeproTech	Cat# 100-39H
Recombinant MurineBMP-4	PeproTech	Cat# 315-27
SB431542	Selleckchem	Cat# S1067
LDN193189	Stemgent	Cat# 04-0074
VEGF165 Protein, Human	MCE	Cat# HY-P78229
Rosiglitazone	Sigma-Aldrich	Cat# R2408
3-Isobutyl-1-methylxanthine (IBMX)	Sigma-Aldrich	Cat# I5879
Dexamethasone (Dex)	Sigma-Aldrich	Cat# D4902
Bovine Insulin	Sigma-Aldrich	Cat# I6634
Bovine Serum Albumin (BSA)	Sigma-Aldrich	Cat# A1470
Ascorbic Acid (Vc)	Sigma-Aldrich	Cat# A4544
KnockOut Serum Replacement	Thermo Fisher Scientific	Cat# A3181502
Neurobasal™ Medium	Thermo Fisher Scientific	Cat# 21103-049
DMEM/F12, GlutaMAX™ supplement	Thermo Fisher Scientific	Cat# 10565-018
N-2 Supplement (100 ×)	Thermo Fisher Scientific	Cat# 17502-048
B-27 ™ Supplement (50 ×), minus vitamin A	Thermo Fisher Scientific	Cat# 12587-010
Insulin–transferrin–selenium (ITS)	Thermo Fisher Scientific	Cat# 41400-045
GlutaMAX™ Supplement	Thermo Fisher Scientific	Cat# 35050-061
2-Mercaptoethanol	Thermo Fisher Scientific	Cat# 21985-023
MEM Non-Essential Amino Acids Solution (100 ×)	Thermo Fisher Scientific	Cat# 1140-050
Penicillin-Streptomycin (10,000 U/mL)	Thermo Fisher Scientific	Cat# 15140-122
Gelatin (0.1 % in water)	Stem Cell Technologies	Cat# 07903
Trypsin-EDTA (0.05%), phenol red	Gibco	Cat# 25300120
DMEM, high glucose, no glutamine	Gibco	Cat# 11960-044
Fetal bovine serum (FBS)	Gibco	Cat# 16000-044
Accutase cell dissociation reagent	Gibco	Cat# A11105-01
TrypLE™ Express	Gibco	Cat# 12605010
Collagenase II	Coolaber	Cat# CC3791G
Mitomycin C	Selleckchem	Cat# S8146
Red Blood Cell Lysis Buffer	Solarbio	Cat# R1010
Dulbecco's phosphate-buffered saline (DPBS)	Gibco	Cat# C14190500CP

Hanks' Balanced Salt Solution (HBSS, with Ca^{2+} & Mg^{2+})	Beyotime	Cat# C0219
Oil Red O Solution	Sigma-Aldrich	Cat# O1391
KaryoMAX Colcemid Solution	Gibco	Cat# 15210-040
Tween 20	Solarbio	Cat# T8220
Triton X-100	Solarbio	Cat# T8200
Matrigel	Corning	Cat# BD354230
Sakura Tissue-Tek® O.C.T. Compound	Sakura	Cat# 4853
Haematoxylin	Sigma-Aldrich	Cat# MHS16
Eosin	Sigma-Aldrich	Cat# HT110116
RNApre pure Cell / Bacteria Kit	TIANGEN	Cat# DP430
Hifair® III 1st Strand cDNA Synthesis SuperMix for qPCR (gDNA digester plus)	YEASEN	Cat# 11141ES60
2 × RealStar Green Power Mixture	GenStar	Cat# A311-05
Alkaline Phosphatase Detection Kit	Millipore	Cat# SCR004
Rapid Giemsa Staining kit	BBI Life Science	Cat# E6073141
Calcein / PI Cell Activity and Cytotoxicity Assay Kit	Beyotime	Cat# C2015M
TIANSeq mRNA capture kit	TIANGEN	Cat# NR105

Supplementary Table 2 Culture medium

(1) pgEpiSCs culture medium

Basic culture medium (BM): 227.5 mL DMEM/F12, 227.5 mL Neurobasal, 2.5 mL N2 supplement, 5 mL B27 supplement, 0.5% GlutaMAX, 1% nonessential amino acids (NEAA), 0.1 mM β-mercaptoethanol, 1% penicillin–streptomycin (PS), 5% knockout serum replacement (KOSR), and 50 μg/mL ascorbic acid.

3i/LAF medium: small molecules and cytokines were added to BM to the following final concentrations, 1 Mm CHIR99021, 2.5 μM IWR-1-endo, 1 μM WH-4-023, 10 ng/mL recombinant human LIF, 25 ng/mL recombinant human activin A, 10 ng/mL recombinant human FGF2.

(2) pFAPs culture and differentiation medium

pFAPs culture medium: DMEM/F12 supplemented with 1% penicillin-streptomycin (PS), 1% nonessential amino acids (NEAA), 10% foetal bovine serum (FBS), and 5 ng/mL FGF2.

pFAPs differentiation medium: DMEM/F12 supplemented with 1% PS, 1% NEAA, 10% FBS, 500μM 3-isobutyl-1-methylxanthine (IBMX), 1 μM Dexamethason (Dex), 10 μg/mL insulin.

pFAPs maintenance medium: DMEM/F12 supplemented with 1% PS, 1% NEAA, 10% FBS, 500μM IBMX, 1 μM Dex, 10 μg/mL insulin.

(3) Adipogenic differentiation medium (ADM)

ADM basic medium (ADM BM): DMEM/F12 supplemented with 1% PS, 1% NEAA, 0.1 mM β-mercaptoethanol, 15% knockout serum replacement (KOSR), and 200 μM ascorbic acid.

ADM I: ADM BM supplemented with 1% B27, 3 μM CHIR99021 and 2 μM SB431542.

ADM II: ADM BM supplemented with 3 μM CHIR99021, 0.5 μM LDN193189 and 20 ng/mL FGF2.

ADM III: ADM BM supplemented with 10ng/mL FGF2 and 10ng/mL hEGF.

ADM IV: ADM BM supplemented with 1% Insulin–transferrin–selenium (ITS), 1 μm Rosiglitazone and Adipogenic Cocktail (500μM IBMX, 1 μM Dex, 10 μg/mL insulin)

ADM V: ADM BM supplemented with 10 μg/mL insulin.

(4) Vascular cells differentiation medium (VDM)

VDM basic medium (VDM BM) is DMEM/F12 supplemented with 1% PS, 1% NEAA, 0.1 mM β-mercaptoethanol, 15% KOSR, and 200 μM ascorbic acid.

VDM I: VDM BM supplemented with 1% B27, 3 μM CHIR99021, 25 ng/mL Activin A, 30 ng/mL BMP4, 50 ng/mL VEGF.

VDM II: VDM BM supplemented with 10 μM SB431542, 50 ng/mL VEGF.

VDM III: VDM BM supplemented with 25 ng/mL VEGF, 10 ng/mL FGF2.

(5) Myogenic differentiation medium (MDM)

MDM basic medium (MDM BM): DMEM/F12 supplemented with 1% NEAA, 0.1 mM β-mercaptoethanol, 1% PS, 15% KOSR, 200 μM ascorbic acid.

MDM I: MDM BM supplemented with 1% B27, 3 μM CHIR99021 and 2 μM SB431542.

MDM II: MDM BM supplemented with 3 μM CHIR99021, 0.5 μM LDN193189, and 20 ng/ml FGF2.

MDM III: MDM BM supplemented with 10 ng/ml HGF, 10 ng/ml IGF-1, 20 ng/ml FGF2 and 0.5 μM LDN193189.

MDM IV: MDM BM supplemented with 10 ng/ml IGF-1.

MDM V: MDM BM supplemented with 10 ng/ml HGF and 10 ng/ml IGF-1.

N2 medium: DMEM/F12 supplemented with 15% KOSR, 1% N2, 1% penicillin–streptomycin, and 1% nonessential amino acids.

(6) Muscle/Adipocyte spheroids differentiation medium (MADM)

MADM basic medium (MADM BM): DMEM/F12 supplemented with 1% PS, 1% NEAA, 0.1 mM β-mercaptoethanol, 15% KOSR, and 200 μM ascorbic acid.

MADM I: MADM BM supplemented with 10 ng/ml IGF-1, 10 ng/ml FGF2, 10 ng/ml hEGF.

MADM II: MADM BM supplemented with 10 ng/ml IGF-1, 10 ng/ml HGF, 500μM IBMX, 1μM Dex, 10 μg/mL insulin.

MADM III: MADM BM supplemented with IGF-1, 10 ng/ml HGF, 10 μg/mL insulin.

(7) Muscle/Adipocyte/Vascular spheroids differentiation medium (MAVDM)

MAVDM basic medium (MAVDM BM): DMEM/F12 supplemented with 1% PS, 1% NEAA, 0.1 mM β-mercaptoethanol, 15% KOSR, and 200 μM ascorbic acid.

MAVDM I: MAVDM BM supplemented with 10 ng/ml IGF-1, 10 ng/ml FGF2, 10 ng/ml hEGF, 50 ng/mL VEGF.<br>MAVDM II: MAVDM BM supplemented with 10 ng/ml IGF-1, 10 ng/ml HGF, 500μM IBMX, 1μM Dex, 10 μg/mL insulin, 50 ng/mL VEGF.<br>MAVDM III: MAVDM BM supplemented with IGF-1, 10 ng/ml HGF, 10 μg/mL insulin, 25 ng/mL VEGF.

Supplementary Table 3 Primer

Gene	SOURCE	IDENTIFIER
OCT4<br>F: CAAACTGAGGTGCCTGCCCTTC<br>R: ATTGAACTTCACCTTCCCTCCA	This paper	N/A
SOX2<br>F: CATCAACGGTACACTGCCTCTC<br>R: ACTCTCCTCCCATTTCCCTCTT	This paper	N/A
NANOG<br>F: CATCTGCTGAGACCCTCGAC<br>R: GGGCTTGTGGAAGAATCAGG	This paper	N/A
T<br>F: GCCAGATCATGCTGAACTCCTTA<br>R: ATAAGCCGTCACCGCTATGAAC	This paper	N/A
PAX6<br>F: TGTCCAACGGATGTGTGAGT<br>R: TCTGTCTCGGATTTCCCAA	This paper	N/A
SOX1<br>F: AGAGGGTACCATTTGCACGG<br>R: AGGGCCAGCCTACAGAACTA	This paper	N/A
GATA6<br>F: CACTACTTGTGCAACCGCTG<br>R: TTCTGCGGCTTTATGAGGGG	This paper	N/A
PAX7<br>F: GTGCCCTCAGTGAGTTCGATTA<br>R: TTCCCTTTGTCGCCCAAGAT	This paper	N/A
MYOD<br>F: CGCTTGAGCAAAGTCAACGA<br>R: GCTATAATCCATCATGCCGTCG	This paper	N/A
MYOG<br>F: CCAGGGGATCATCTGCTCACA<br>R: TGGGCATGGTTTCATCTGGG	This paper	N/A
MYMK<br>F: CTTCCTCCCCACGGTCAG<br>R: TACTCCAGGATGTCAAGGCG	This paper	N/A
MYH2<br>F: GGGCTCAAACTGGTGAAGC<br>R: AGATGCGGATGCCCTCCA	This paper	N/A
MYH3<br>F: GCCGACGCTGACAGCGGAAA<br>R: AGATGCGGATGCCCTCCA	This paper	N/A
MYH11<br>F: GAGCGCCACATCTCAACTCT<br>R: CTCCTCGGCCAACAACTGAT	This paper	N/A
PDGFRA<br>F: ATCGTGGAGAATCTGCTGCCTG<br>R: GATGATGTAGCCGCTGTCTG	This paper	N/A
VIM<br>F: GCCCGTCACCTTCGTGAATA<br>R: GTCCATCTCTGGTCTCAACCG	This paper	N/A
DLK1<br>F: GTCTGTGCAAGCCCAAGTTC<br>R: GTTGTAGCGGAGGTTGGACA	This paper	N/A

ZNF423<br>F: AAGCTTCTAAGCGAGCAGGC<br>R: CCTCCCAGCTCGATGGTTTT	This paper	N/A
ITGB1<br>F: CCAAATGGGACACGGGTGA<br>R: AGCTACCTCACTGTGACTGC	This paper	N/A
THY1<br>F: GGCATCGCTCTCTTGCTAAC<br>R: GGACCTTGATGTCGTACTTGC	This paper	N/A
ENG<br>F: ATGCTGTCGTAGCAACCCAA<br>R: GCCGGACCTCTTCTGTTCTC	This paper	N/A
PTPRC<br>F: TGATGATTGCTGCTCAGGGG<br>R: CTCTTCCGCATTCCAGTGGT	This paper	N/A
CEBPA<br>F: GGCCAGCACACACACATTAGA<br>R: CCCCCAAAGAAGAGAACCAAG	This paper	N/A
PPARG<br>F: GAGGGCGATCTTGACAGGAA<br>R: GCCACCTCTTTGCTCTGCTC	This paper	N/A
FABP4<br>F: TGAAAGAAGTGGGAGTGGGC<br>R: CTGGCCCAATTTGAAGGCAA	This paper	N/A
ADIPOQ<br>F: TTGAAGGTCCCCGAGGTTTC<br>R: CCACACTGAATGCTGAACGG	This paper	N/A
LEP<br>F: TACGGTTGAATGCCCGTTGA<br>R: TCCATTAGTCTCACGGCAGC	This paper	N/A
PLIN1<br>F: CGATCTCCCTCGTGACTTGG<br>R: ACGTTGTCAGTAACGCCCTT	This paper	N/A
PDGFRB<br>F: TTCTCCCAGCTGAGCCAATC<br>R: CCAAGTGGCTCACAAAACGG	This paper	N/A
CDH5<br>F: AAGAACATCGCCCGTGTCAT<br>R: CACTGAGCCGATCCAAGGTT	This paper	N/A
PECAM1<br>F: CACCGAGGTCTGGGAACAAA<br>R: GGGAGCCTTCCGTTCTAGAATATC	This paper	N/A
COL5A2<br>F: AAACTGGGCAGAAGCAAGAC<br>R: ATTTCTTCACCATATCCTTCATCCT	This paper	N/A
COL3A1<br>F: CTAGCCGAGCTTCCCAGAAC<br>R: TCCCCAGTGTGTTTAGTGCAA	This paper	N/A
EF1A<br>F: AATGCGGTGGGATCGACAAA<br>R: CACGCTCACGTTCAGCCTTT	This paper	N/A

Supplementary Table 4 Antibodies

REAGENT or RESOURCE	SOURCE	IDENTIFIER	DILUTION RATIO
Rabbit polyclonal anti-human Nanog	PeproTech	Cat# 500-P236	1:500
Goat polyclonal anti-Brachyury	Santa Cruz Biotechnology	Cat# sc 17743	1:50
Rabbit polyclonal anti-MYOD1	Proteintech	Cat# 18943-1-AP	1:200
Mouse monoclonal anti-Skeletal Myosin (Fast)	Sigma-Aldrich	Cat# M4276	1:300
Mouse monoclonal anti-Myosin heavy chain (MyHc)	DSHB	Cat# MF20-S	1:200
Mouse PDGFR alpha	R&D Systems	Cat# AF1062-SP	1:300
Rabbit polyclonal Integrin Beta 1 (CD29)	Proteintech	Cat# 12594-1-AP	1:300
Mouse monoclonal CD45 (35-Z6)	Santa Cruz Biotechnology	Cat# sc-1178	1:300
Rabbit FABP4 Polyclonal	Proteintech	Cat# 12802-1-AP	1:300
Rabbit polyclonal eNOS	MCE	Cat# HY-P80656	1:300
Rabbit polyclonal Anti-CD31	abcam	Cat# ab28364	1:300
Rabbit polyclonal anti-alpha smooth muscle Actin	Abcam	Cat# ab5694	1:250
Human DiI-Acetylated Low Density Lipoprotein (Human DiI-Ac-LDL)	YEASEN	Cat# 20606ES76	1:200
Rabbit COL4A4 Polyclonal antibody	Proteintech	19674-1-AP	1:300
Nile Red	Sigma-Aldrich	Cat# 19123	1:100
Hoechst 33258	Beyotime	Cat# C1025	1:100
BODIPY	Duofluor	Cat# P10051-100	1:100
Actin-Tracker Red-594	Beyotime	Cat# C2205S	1:100
DAPI	Roche Life Science	Cat# 10236276001	1:10000
Donkey anti-Rabbit IgG (H+L) highly Cross-Adsorbed Secondary Antibody, Alexa Fluor™ 594	Invitrogen	Cat# A-21207	1:1000
Donkey anti-Mouse IgG (H+L) highly Cross-Adsorbed Secondary Antibody, Alexa Fluor™ 594	Invitrogen	Cat# A-21203	1:1000
Donkey anti-Goat IgG (H+L) Cross-Adsorbed Secondary Antibody, Alexa Fluor™ 594	Invitrogen	Cat# A-11058	1:1000
Donkey anti-Mouse IgG (H+L) Highly Cross-Adsorbed Secondary Antibody, Alexa Fluor™ 488	Invitrogen	Cat# A-21202	1:1000
Donkey anti-Mouse IgG (H+L) Highly Cross-Adsorbed Secondary Antibody, Alexa Fluor™ Plus 647	Invitrogen	Cat# A32787	1:1000

Supplementary Table 5 The recipe for sausages			
**Ingredients**	**Conventional meat (%)**	**pgEpiSCs meat (%)**	**Cultured meat (%)**
Pork (fat : lean = 2.5:7.5)	75.00	50.00	50.00
pgEpiSCs	-	25.00	-
Cultured meat	-	-	25.00
Edible salt	1.60	1.60	1.60
Sodium tripolyphosphate	0.35	0.35	0.35
Ice water	15.00	15.00	15.00
Collagen	2.00	2.00	2.00
White pepper powder	0.12	0.12	0.12
Five-spice powder	0.05	0.05	0.05
Corn starch	5.00	5.00	5.00
Monascus red	0.012	0.012	0.012
White sugar	1.20	1.20	1.20
Total	100.332	100.332	100.332
