# Supplementary material for: Generation of multitissue cell-cultivated meat via multidirectional differentiation of stable porcine epiblast stem cells
Source: Nat Commun. 2026 Mar 2;17:3347. doi: 10.1038/s41467-026-70177-w (PMC13066626; doi:10.1038/s41467-026-70177-w)
Supplement: Supplementary file 2 — Reporting Summary [file 41467_2026_70177_MOESM2_ESM.pdf]

nature portfolio

Corresponding author(s): Jianyong Han, Suying Cao, Aijin Ma.

Last updated by author(s): Jan 30, 2026

Reporting Summary

Nature Portfolio wishes to improve the reproducibility of the work that we publish. This form provides structure for consistency and transparency in reporting. For further information on Nature Portfolio policies, see our Editorial Policies and the Editorial Policy Checklist.

Statistics

For all statistical analyses, confirm that the following items are present in the figure legend, table legend, main text, or Methods section.

n/a	Confirmed	
☐	☒	The exact sample size (*n*) for each experimental group/condition, given as a discrete number and unit of measurement
☐	☒	A statement on whether measurements were taken from distinct samples or whether the same sample was measured repeatedly
☐	☒	The statistical test(s) used AND whether they are one- or two-sided<br>*Only common tests should be described solely by name; describe more complex techniques in the Methods section.*
☒	☐	A description of all covariates tested
☐	☒	A description of any assumptions or corrections, such as tests of normality and adjustment for multiple comparisons
☐	☒	A full description of the statistical parameters including central tendency (e.g. means) or other basic estimates (e.g. regression coefficient) AND variation (e.g. standard deviation) or associated estimates of uncertainty (e.g. confidence intervals)
☐	☒	For null hypothesis testing, the test statistic (e.g. *F*, *t*, *r*) with confidence intervals, effect sizes, degrees of freedom and *P* value noted<br>*Give P values as exact values whenever suitable.*
☒	☐	For Bayesian analysis, information on the choice of priors and Markov chain Monte Carlo settings
☒	☐	For hierarchical and complex designs, identification of the appropriate level for tests and full reporting of outcomes
☒	☐	Estimates of effect sizes (e.g. Cohen's *d*, Pearson's *r*), indicating how they were calculated

Our web collection on statistics for biologists contains articles on many of the points above.

Software and code

Policy information about availability of computer code

Data collection	The data of RT-PCR was collected Archimed X6(ROCGENE, China).<br>The data of morphology of cells on scaffolds, spheroids and fluorescence were collected by inverted microscopy (OLYMPUs, CKx41), scanning electron microscopy (HITACHI, TM-4000 plus).<br>The data of cell viability was collected by BD FACSVerse (BD Biosciences, USA).<br>The images of cells on 3D edible scaffolds and tissue sections of spheroids were taken using a laser scanning confocal microscope (Lecai STELLARIS5, Germany).<br>Texture profile analysis (TPA) of spheroids was measured by a texture analyzer (TA. XT Plus, Stable Micro system Lte, UK).<br>The determination of individual amino acids by using a fully automated amino acid analyzer (LA8080, Hi-tach, Tokyo, Japan) for comparison with the standard (013-08391. Wako, Tokyo, Japan).<br>The composition determination of fatty acids was accomplished by a gas chromatograph-mass spectrometer (Trace1310 15Q, ThermoFisher, USA) and compared with the standard.<br>The detection of flavor substances in cell-cultured meat was accomplished by gas chromatograph - Mass spectometer (7890B-7000C, Agilnt Technologies, USA).<br>The genetic information and qPCR primer desige were from NCBI GEO.
Data analysis	ImageJ; GraphPad Prism (v10); FlowJo (v10); FastQC (v0.11.9); Trimmomatic (v0.39); Kallisto (v0.46.0); R (v4.3.0); tximport R package (v1.18.0); DESeq2 R package (v1.30.0); ggplot2 R package (v3.4.0); pheatmap R package (v1.0.12); fgsea R package (v1.26.0); Metascape (web-based tool, v3.5, accessed 2024-09-01); Adobe Illustrator CC 2019.

For manuscripts utilizing custom algorithms or software that are central to the research but not yet described in published literature, software must be made available to editors and reviewers. We strongly encourage code deposition in a community repository (e.g. GitHub). See the Nature Portfolio guidelines for submitting code & software for further information.

Data

Policy information about availability of data

All manuscripts must include a data availability statement. This statement should provide the following information, where applicable:

- Accession codes, unique identifiers, or web links for publicly available datasets
- A description of any restrictions on data availability
- For clinical datasets or third party data, please ensure that the statement adheres to our policy

Provide your data availability statement here.

Research involving human participants, their data, or biological material

Policy information about studies with human participants or human data. See also policy information about sex, gender (identity/presentation), and sexual orientation and race, ethnicity and racism.

Reporting on sex and gender	N/A
Reporting on race, ethnicity, or other socially relevant groupings	N/A
Population characteristics	N/A
Recruitment	N/A
Ethics oversight	N/A

Note that full information on the approval of the study protocol must also be provided in the manuscript.

Field-specific reporting

Please select the one below that is the best fit for your research. If you are not sure, read the appropriate sections before making your selection.

- [x] Life sciences
- [ ] Behavioural & social sciences
- [ ] Ecological, evolutionary & environmental sciences

For a reference copy of the document with all sections, see nature.com/documents/nr-reporting-summary-flat.pdf

Life sciences study design

All studies must disclose on these points even when the disclosure is negative.

Sample size	Intial sample size per experiment was determined based on preliminary studies, and were diferent for different types of assays, as necessary to achieve statistical significance for data. Experiments were repeated multiple times in triplicate samples unless indicated otherwise.
Data exclusions	No data were excluded from the analyses.
Replication	The experiments were replicated or performed independently based on the numbers mentioned in the manuscript (5uch as methods sectionand figure legends).
Randomization	There were no clinical populations or patients in this studly, therefore randomization techniques were not applicable. The same well plate theexperiment was conducted in by dividing into treatments at random, which collects images of samples and experimental data.
Blinding	Investigators were blinded to group allocation during [data collection/analysis/both]. Group identities were coded by an independent researcher, and the codes were not revealed until after [data collection/statistical analysis was completed.

Reporting for specific materials, systems and methods

We require information from authors about some types of materials, experimental systems and methods used in many studies. Here, indicate whether each material, system or method listed is relevant to your study. If you are not sure if a list item applies to your research, read the appropriate section before selecting a response.

Materials & experimental systems

n/a	Involved in the study
	☒ Antibodies
	☒ Eukaryotic cell lines
☒	☐ Palaeontology and archaeology
	☒ Animals and other organisms
☒	☐ Clinical data
☒	☐ Dual use research of concern
☒	☐ Plants

Methods

n/a	Involved in the study
☒	☐ ChIP-seq
	☒ Flow cytometry
☒	☐ MRI-based neuroimaging

Antibodies

Antibodies used

Primary antibodies:
Rabbit polyclonal anti-human Nanog, PeproTech, 500-P236, 1:500
Goat polyclonal anti-Brachyury, Santa Cruz Biotechnology, sc17743, 1:50
Rabbit polyclonal anti-MYOD1, Proteintech, 18943-1-AP, 1:200
Mouse monoclonal anti-Skeletal Myosin (fast), Sigma-Aldrich, M4276, 1:300
Mouse monoclonal anti-Myosin heavy chain (MyHc), DSHB, MF20-S, 1:200
Mouse PDGFR alpha, R&D Systems, AF1062-SP, 1:300
Rabbit polyclonal Integrin Bera 1 (CD29), Proteintech, 12594-1-AP, 1:300
Mouse monoclonal CD45 (35-Z6), Santa Cruz Biotechnology, sc-1178, 1:300
Rabbit FABP4 Polyclonal, Proteintech, 12802-1-AP, 1:300
Rabbit polyclonal eNOS, MCE, HY-P80656, 1:300
Rabbit polyclonal Anti-CD31, Abcam, ab28364, 1:300
Rabbit polyclonal anti-alpha smooth muscle Actin, Abcam, ab5694, 1:250
Rabbit COL4A4 Polyclonal antibody, Proteintech, 19674-1-AP, 1:300
Human Dil-Acetylated Low Density Lipoprotein (Human Dil-Ac-LDL), YEASEN, 20606ES76, 1:200
Secondary antibodies:
Donkey anti-Rabbit IgG (H+L) highly Cross-Adsorbed Secondary Antibody, Alexa Fluor 594, Invitrogen, A-21207, 1:1000
Donkey anti-Mouse IgG (H+L) highly Cross-Adsorbed Secondary Antibody, Alexa Fluor 594, Invitrogen, A-21203, 1:1000
Donkey anti-Goat IgG (H+L) Cross-Adsorbed Secondary Antibody, Alexa Fluor 594, Invitrogen, A11058, 1:1000
Donkey anti-Mouse IgG (H+L) Highly Cross-Adsorbed Secondary Antibody, Alexa Fluor 488, Invitrogen, A-21202, 1:1000
Donkey anti-Mouse IgG(H+L) Highly Cross-Adsorbed Secondary Antibody, Alexa Fluor Plus 647, Invitrogen, A32787, 1:1000

Validation

Antibodies associated of pluripotency were validated in the previous work (Zhi et al., 2022, http:// doi.org/10.1038/s41422-021-00592-9). Antibodies associated of myogenesis were validated in the previous work (Zhu et al., 2023, https:// doi.org:10.1038/s41467-023-44001-8). Antibodies associated of adipogenesis were validated on procine fibro/adipogenic progenitors in this study.
Rabbit polyclonal anti-human Nanog (https://www.peprotech.com/zh/anti-human-nanog)
Goat polyclonal anti-Brachyury (https://www.scbt.com/p/brachyury-antibody-n-19?requestFrom=search)
Rabbit polyclonal anti-MYOD1 (https://www.ptgcn.com/products/MYOD1-Antibody-18943-1-AP.htm)
Mouse monoclonal anti-Skeletal Myosin (Fast) (https://www.sigmaaldrich.cn/CN/zh/product/sigma/m4276)
Mouse monoclonal anti-Myosin heavy chain (MyHc) (https://dshb.biology.uiowa.edu/MF-20)
Mouse PDGFR alpha (https://www.rndsystems.com/products/mouse-pdgf-ralpha-antibody_af1062)
Rabbit polyclonal Integrin Bera 1 (CD29) (https://www.ptgcn.com/Products/ITGB1-Antibody-12594-1-AP.htm)
Mouse monoclonal CD45 (35-Z6) (https://www.scbt.com/p/cd45-antibody-35-z6)
Rabbit FABP4 Polyclonal (https://www.ptgcn.com/Products/FABP4-Antibody-12802-1-AP.htm)
Rabbit polyclonal eNOS (https://www.medchemexpress.cn/antibody/enos-rabbit-pab.html)
Rabbit polyclonal Anti-CD31 (https://www.abcam.com/en-us/products/primary-antibodies/cd31-antibody-ab28364)
Rabbit COL4A4 Polyclonal antibody (https://www.ptgcn.com/Products/COL4A4-Specific-Antibody-19674-1-AP.htm)
Actin-Tracker Red-594 (https://www.beyotime.com/product/C2205S.htm)
Donkey anti-Rabbit IgG (H+L) highly Cross-Adsorbed Secondary Antibody, Alexa Fluor 594 (https://www.thermofisher.cn/cn/zh/antibody/product/Donkey-anti-Rabbit-IgG-H-L-Highly-Cross-Adsorbed-Secondary-Antibody-Polyclonal/A-21207)
Donkey anti-Mouse IgG (H+L) highly Cross-Adsorbed Secondary Antibody, Alexa Fluor 594 (https://www.thermofisher.cn/cn/zh/antibody/product/Donkey-anti-Mouse-IgG-H-L-Highly-Cross-Adsorbed-Secondary-Antibody-Polyclonal/A-21203)
Donkey anti-Mouse IgG (H+L) highly Cross-Adsorbed Secondary Antibody, Alexa Fluor 488 (https://www.thermofisher.cn/cn/zh/antibody/product/Donkey-anti-Mouse-IgG-H-L-Highly-Cross-Adsorbed-Secondary-Antibody-Polyclonal/A-21202)
Donkey anti-Goat IgG (H+L) Cross-Adsorbed Secondary Antibody, Alexa Fluor 594 (https://www.thermofisher.cn/cn/zh/antibody/product/Donkey-anti-Goat-IgG-H-L-Cross-Adsorbed-Secondary-Antibody-Polyclonal/A-21447)
Donkey anti-Mouse IgG(H+L) Highly Cross-Adsorbed Secondary Antibody, Alexa Fluor Plus 647 (https://www.thermofisher.cn/cn/zh/antibody/product/Donkey-anti-Mouse-IgG-H-L-Highly-Cross-Adsorbed-Secondary-Antibody-Polyclonal/A-31571)

Eukaryotic cell lines

Policy information about cell lines and Sex and Gender in Research

Cell line source(s)	The pgEpisCs were derived from the embryo of NongDa Xiang pig in the previous work (Zhi et al., 2022, http://doi.org/10.1038/s41422-021-00592-9). The porcine fibro/adipogenic progenitors cell line was isolated in this study and described within.
Authentication	The pgEpiSCs were authenticated by whole genome sequencing. The porcine fibro-adipogenic progenitors cells authenticated by routinely by morphology, PCR, RT-PCR and immunofluorescence staining.
Mycoplasma contamination	We confirmed that all cell lines tested were negative for mycoplasma contamination.
Commonly misidentified lines (See ICLAC register)	N/A

Animals and other research organisms

Policy information about studies involving animals; ARRIVE guidelines recommended for reporting animal research, and Sex and Gender in Research

Laboratory animals	ICR mice used for isolation of mouse embryonic fibroblasts (MEFs) were purchased from Beijing SiPeiFu Biotechnology CO.,Ltd (Belijing, China), All mice were individually housed under a 12r light/dark cycle in a sterile environment and provided with food and water ad libitum. The ambient temperature for housing mice in this study was maintained at 21-26°C, with relative humidity controlled at 50-60%. The Nongda Xiang pig (1-week-old) was used for the isolation of porcine fibro/adipogenic progenitors.
Wild animals	N/A
Reporting on sex	Female ICR mice were used to isolate mouse embryonic fibroblasts (MEFs). Pig was only used to isolate fibro/adipogenic progenitors and did not invole sex-and gender-based analysis
Field-collected samples	N/A
Ethics oversight	All the mouse and pig experiments performed were approved by the Institutional Animal Care and Use Committee of China Agricultural University (AW 52114202-3-01).

Note that full information on the approval of the study protocol must also be provided in the manuscript.

Plants

Seed stocks	*Report on the source of all seed stocks or other plant material used. If applicable, state the seed stock centre and catalogue number. If plant specimens were collected from the field, describe the collection location, date and sampling procedures.*
Novel plant genotypes	*Describe the methods by which all novel plant genotypes were produced. This includes those generated by transgenic approaches, gene editing, chemical/radiation-based mutagenesis and hybridization. For transgenic lines, describe the transformation method, the number of independent lines analyzed and the generation upon which experiments were performed. For gene-edited lines, describe the editor used, the endogenous sequence targeted for editing, the targeting guide RNA sequence (if applicable) and how the editor was applied.*
Authentication	*Describe any authentication procedures for each seed stock used or novel genotype generated. Describe any experiments used to assess the effect of a mutation and, where applicable, how potential secondary effects (e.g. second site T-DNA insertions, mosiacism, off-target gene editing) were examined.*

Flow Cytometry

Plots

Confirm that:

- [x] The axis labels state the marker and fluorochrome used (e.g. CD4-FITC).
- [x] The axis scales are clearly visible. Include numbers along axes only for bottom left plot of group (a 'group' is an analysis of identical markers).
- [x] All plots are contour plots with outliers or pseudocolor plots.
- [x] A numerical value for number of cells or percentage (with statistics) is provided.

Methodology

Sample preparation	Cell survival status was measured by using the Calcein/PI Cell Activity and Cytotoxicity Assay Kit. Cells were stained with Calcein-AM (AM) and propidium iodide (PI) double fluorescence, observed with a fluorescence microscope, and

flowcytometry was used to determine cell survival effectiveness, where unstained cells were used as negative controls fordelineation of FACS gating parameters.

Instrument	BD FACSVerse (BD Biosciences, USA).
Software	The FlowJo_v10.8.1_CL software was used in FACS data analysis.
Cell population abundance	The Calcein-AM is characterized as a living cells. The ropidium iodide (PI, red) is characterized as dead cells.<br>For each sample, ~1 × 10^6 viable cells are needed.
Gating strategy	The gating strategy is exemplified in Fig, 2,3 and Supplementary Fig. 4, 5, 6, 9. Flow cytometric analysis Cells were digested by TryPLE for 5 min into single cells and centrifuged at 1500 × g for 5 min, which were washed three times with DPBS before being resuspended with diluted fluorochrome and incubated at 37°C for 30min. For each sample, ~1 × 10^6 viable cells are needed. Following incubation, cells may be analyzed directly by flow cytometry. Calcein AM fluoresces green (excitation/emission maxima: 494/517 nm), whereas PI emits red fluorescence (excitation/emission maxima: 535/617 nm). All procedures must be conducted under low-light or dark conditions. The unstained cells suspended only in the assay buffer and not exposed to any fluorescent dyes were used to control.

☒ Tick this box to confirm that a figure exemplifying the gating strategy is provided in the Supplementary Information.
